# Hydrotherapy in Appendicitis1Read before the Central New York Medical Association, Buffalo, N. Y., October 16, 1894.

**Published:** 1894-12

**Authors:** George E. Clark

**Affiliations:** Skaneateles, N. Y.


### HYDROTHERAPY IN APPENDICITIS.[1]

By GEORGE E. CLARK, M. D., Skaneateles, N. Y.

Hydrotherapy in the treatment of appendicitis is by no means new, but I feel sure the subject has not yet received due attention.

From the opinions expressed by more than twenty physicians writing upon this subject in our journals, and from a perusal of the articles upon appendicitis in five recent text-books of medicine, I gather that the use of water is sometimes ignored, sometimes condemned and never mentioned favorably, except in the most casual way, when it is advised for the purpose of moving the bowels.

*This*, I maintain, is *not* doing it justice!

I shall endeavor to show you, by a report of seven consecutive cases treated and cured by the use of water alone, that there is more in hydrotherapy than the simple moving of the bowels. Osler, of Baltimore, says: "Briefly stated, localized pain in the right iliac fossa, with or without induration or tumor, the existence of McBurney's tender point, fever, furred tongue, vomiting, constipation or diarrhea, indicate appendicitis."

All of my cases had localized pain in the right iliac fossa, induration or tumor, McBurney's tender point, fever and furred tongue. Some had vomiting, all had diarrhea or constipation, or diarrhea followed by constipation.

The treatment, in each case, was as follows: Cloths, about four inches square, wet in cold water, were applied to the right iliac region and changed, if necessary, every three to five minutes, until the pain was completely relieved. Cold, *not warm*, water enemata were given every one to three hours, night and day, according to the severity of the case, and continued until the cure was complete. In some cases, an initial dose of phenacetine was given, where the temperature was high, but beyond this, no medicinal treatment whatever was adopted. The patients were all put upon a liquid diet until convalescent. The following is a brief synopsis of the cases:

Case I.—F. B., farmer, *æt.* 46. The attack followed severe effort in lifting accompanied with a blow from the handle of a plow. About twelve hours later he became conscious of pain in the right iliac fossa, which soon became severe and was accompanied with excessive tenderness; so much so that he could not straighten up. When seen by me

1. Read before the Central New York Medical Association, Buffalo, N. Y., October 16, 1894.

the next day he was in bed, lying on his back with the thigh flexed, complaining of pain on the slightest motion. Temperature was 100½° F., and the pulse not much elevated. Examination revealed an indurated area in the right iliac fossa, the McBurney point very tender, the abdomen neither distended nor tender, except in the region of the appendix; here, however, the least pressure caused great pain. The bowels were constipated, but there had been no vomiting.

Cold water externally and cold water enemata, at first every four hours and later three times daily, brought the temperature to normal in forty-eight hours. The induration and tenderness persisted for several days, but grew constantly less. For some time after, the man noticed a tearing sensation whenever an extra strain was put upon the muscles in that region, but there has been no second attack. I paid my first visit to this patient on the 15th day of the month, and my last on the 21st day—six days' treatment in all.

Case II.—Mrs. H., *æt.* 23, June 24th, was seized with acute pain in right iliac region, seeming to shoot down towards the bladder. The temperature was normal, there was no tumor or induration, and very little tenderness on pressure. I made a diagnosis of renal colic and relieved the pain promptly by hypodermic injection of morphia. I was called again the same day and was again obliged to resort to the hypodermic needle. On calling the next day I found the patient not yet well, but so much better that I did not visit her on the following day. June 27th, the fourth day from my first visit, I was called once more. I found the patient suffering great pain in the right iliac region. There was a decided induration and excessive tenderness, McBurney's point being well marked. The temperature was 103° F., the tongue furred and the bowels constipated. I began the cold water external applications at once, but she had no enemata until her mother arrived in the evening, after which time they were given every three hours. About forty-eight hours later the temperature was normal, the pain in the meantime having quite gone, and the induration had almost, if not entirely, disappeared. In this case a quantity of seeds came away with the enemata. I made my last visit July 2d, nine days after the symptoms, which I diagnosed as renal colic, five days after the positive diagnosis of appendicitis.

Case III.—Walter L., *æt.* 14, on the afternoon of September 25th began to have pain on right side of the abdomen, which steadily increased until early next morning, by which time it had become excessive and was accompanied by vomiting and diarrhea. At this time I was called. I found the boy lying with the right thigh flexed, holding the bed clothes away from his side with the hand. The tenderness was so great that the mere approach of the hand would make him flinch. The whole abdomen seemed tender, but this was probably owing to the disturbance pressure caused in the iliac region. All the classical signs of appendicitis were present; the temperature was 105° F. There was a delay of a few hours in getting assistance, and, in the meantime, small doses of phenacetine were given to reduce the temperature. In this case the enemata were given at first every hour. In forty-eight hours from my first visit the temperature was normal, the tenderness almost gone and very little induration left. My last visit was made on the 28th, three days after my first call, and two days later the boy rode five miles over a very rough road without any pain or inconvenience.

CASE IV.—This was a second attack occurring to the boy just mentioned. On January 14th, about four months later you will observe, after an afternoon's skating, he was taken with a repetition of his previous symptoms. Profiting by former experience, his treatment was at once started, and by the time I arrived, several hours later, he was somewhat relieved. His temperature was then 102° F., the induration was slight, if present at all; there was considerable pain; he had vomited and his bowels were constipated. No medicine was given. I made him three visits. He was well on the 16th, *i.e.*, in three days. I learn that he has since had one slight attack, but found it unnecessary to send for me.

CASE V.—Miss R., *æt.* 18, was a case seen in consultation. She was taken ill September 24th with a chill, vomiting, diarrhea, fever, and pain in abdomen. Her physician was called on the following day. Within a short time she developed a decided tumor in the right iliac region, accompanied with excessive tenderness, especially over McBurney's point. She was treated with turpentine, morphine, some powders for her nausea and gradually increasing doses of quinine until six grains every two hours were taken. The abdomen was poulticed and the tumor painted with iodine. This treatment was continued for eleven days, the temperature ranging from 102° to 103° during the whole of that time in spite of the large doses of quinine which were given. On October 6th I saw her in consultation, and as her physician was about to start for the World's Fair, she was left in my hands. At this time the temperature was 102½°, the tenderness was great and the tumor could be seen very plainly. It was from three to four inches long and perhaps one and one-half inches wide. No fluctuation could be felt, although on account of the tenderness, deep pressure was not made. A rectal examination was not made. I continued the treatment for the first twenty-four hours. At the end of that time the temperature was one degree higher, and there was in *no* respect a change for the better. I then stopped all the medicine and the poultices. I gave one dose of phenacetine—five grains. I ordered constant cold water applications to the tumor and cold water enemata every two or three hours. I met with some opposition from the family, but I overcame it. At my next visit, twenty-four hours later, that girl's temperature was normal and the tumor was fully half gone. In three days she got out of bed for a few minutes, and she had not a single bad symptom after that. I made her in all but seven visits. She has had no return of the trouble.

CASE VI.—Was also seen in consultation. W. H., carpenter, *æt.* 25. March 6th, at 3.30 P. M., he was taken with pain in right iliac region, accompanied with vomiting and diarrhea. His pain increased rapidly, and his physician was called at 7 P. M. The physician, from these symptoms and the presence of McBurney's point, made a diagnosis of appendicitis. The temperature was, at this time, about 102° F. He was given calomel as a laxative, phenacetine for his fever, and morphine for his pain. The abdomen was poulticed. The patient grew worse, and on March 9th I was called in consultation. At this time, the temperature, in spite of the phenacetine, was 102° F., and I learned that it had ranged from that to 103°. There was marked induration in the iliac region and great tenderness. I could detect no fluctuation. The poultices and the medicines were stopped, cold water applied externally and cold enemata given every hour. The temperature was reduced promptly and the pain and induration disappeared so quickly that, two days later, his physician reported that he was "kicking because he could not get out of bed." His physician made his last visit on March 15th, nine days after the inception of the disease and six days after the water treatment was begun. I have recently seen this patient, and learn that he had some pain, due to adhesions in the right iliac region, which persisted for a week or more after he began to go about; this gradually disappeared and he has since had no trouble whatsoever.

CASE VII.—Was seen after he had been treated for nearly a week by another physician. J. H., *æt.* 19, laborer. Six days before my first visit he was taken ill with fever, accompanied by pain on the right side of the abdomen. The pain gradually increased and was soon accompanied with vomiting and diarrhea. He was treated by a physician, but, as I saw only the patient, I can give no particulars of the treatment further than to say it was medicinal. The patient was not satisfied with his progress and I was called. I found him with a temperature of 103½° F. There was an indurated mass in the right iliac fossa, about the size of a small egg. The tenderness was marked but not excessive. For comfort, the thigh was kept flexed. The tongue was thickly coated and the bowels constipated. The cold water treatment brought his temperature to normal within three days, and his other symptoms had so far subsided by that time that I did not visit him again. He kept to the house for nearly a week longer, because of pain on his making certain movements. After this he went to work on a farm. When I saw him, about two weeks ago, he stated that he had been perfectly well ever since.

In reviewing these cases, it is worthy of note that, taking into consideration the comparative severity of the symptoms and the time which had elapsed between the onset of the disease and the first application of the water, the success of the treatment seems to have been largely in proportion to the frequency of the enemata. For instance, in Case I., where the enemata were given only every four hours and, later, thrice daily, the cure was slow and there was pain from adhesions, lasting for some time after. In Case II., where the enemata were given steadily every three hours, although the symptoms were much more severe, and not at first recognized, the *cure* was more prompt. In Case III., perhaps the most acute of the seven, the enemata were given every hour and the cure was still more prompt. In general, the same reasoning applies to the other cases. In Cases V., VI. and VII., in each of which other treatment had previously been tried, the striking and almost immediate improvement consequent upon the change could not be attributed to anything but the use of the water.

These enemata were not given for the purpose of moving the bowels, although they incidentally had that effect. They were given with the belief that they had some intrinsic influence upon the course of the disease, the proof of this being their greater efficacy when given more frequently—more frequently than could possibly be needed in order to empty the large intestine. Cold reduces temperature not only by the local abstraction of heat, but also by influencing the circulation of the part. It is likely that some of the water finds its way round to the cecum, but I doubt if much good is effected in that way. The upper part of the rectum lies in the median line at the usual level of the appendix, and they cannot be much more than two inches apart. Now, the application of cold water causes, at first, a contraction of the blood-vessels, which is soon followed by a dilatation, the dilatation being much more persistent than the contraction. Why may not this reactionary dilatation withdraw some blood from the region of the appendix and so lessen the inflammation there?

I am *not* a fanatic. I do *not* believe that cold water will cure *every* case of appendicitis. I *do* believe that surgical measures are *sometimes* necessary. I was rooted and grounded in the belief that as soon as the diagnosis could be made, the appendix should be removed. As to that, I have changed my mind. I now believe that *cold* water, used *early* and *freely* and *often*, in the manner indicated, should *first* be tried, and, if the symptoms do not improve within twenty-four hours, surgical measures should be considered.

DISCUSSION.

Dr. N. S. Jacobson: If the committee will allow me a moment, I would like to say a word or two with reference to this paper. Personally, I must take exception to the conclusions. I do not think a case has been established of post hoc propter hoc by any means. As far as the foreign literature is concerned, we have reason to believe that fully 85 or 90 per cent. of all cases of appendicitis are presumed to be cured by medicinal treatment. That is to say, they recover spontaneously under some form of medicinal treatment. But the principle which is at stake is not well borne out, it seems to me, by the line of argument. If cold is to be used in this class of cases, it must be used as an anti-phlogistic, as cold cannot be beneficial in a case, for example, like one of the cases mentioned, which has run along ten or twelve days. Secondly, there is a question as to diagnosis in these cases, as, for instance, in one of the cases mentioned a large number of seeds were discharged. In the third place, there is a marked question as to whether you have your cold in contact with the diseased area. Now, what have we in this class of cases? We have in a group of cases simply a catarrhal indication of appendicitis. In a vast majority of cases you have perforations, ulcerations, you have thrombi existing and other disturbances occurring, and there is no reason to believe that the simple application of cold into the rectum at a remote point, or that the form of application of cold externally which is not accepted as a very good method of cold application,—as compresses externally,—there is no reason to justify us that we can depend upon this class of treatment. This matter is altogether of too much importance to be passed by lightly.

---

Meningitis Due to the Bacillus of Typhoid Fever.—Daddi (*Sperimentale*, Juillet, 1894; *Revue Internat. de Méd. et de Chir. Prat.*, No. 18, p. 335) has reported the case of a boy, nine years old, who in the course of an attack of typhoid fever was seized with symptoms of meningitis, to which he succumbed. In the creamy exudate covering the upper surface of the brain and cerebellum, as well as in two small abscesses situated symmetrically at the level of the spines of the scapula on either side, the bacillus of typhoid fever was found in pure culture.—*Med. News*, November 17, 1894.

